# Comprehensive analysis of the effects of high stocking density stress on broiler health

**DOI:** 10.3389/fvets.2025.1741407

**Published:** 2026-01-07

**Authors:** Dongying Bai, Caifang Guo, Penghui Ma, Ziwei Wang, Xiaodie Zhao, Xiqiang Ma, Xiaolin Xie, Yi Zhang, Fangshen Guo, Yushu Zhang, Bingkun Zhang, Cai Zhang, Wenrui Zhen, Yanbo Ma

**Affiliations:** 1 Department of Animal Physiology, College of Animal Science and Technology, Henan University of Science and Technology, Luoyang, China; 2 Henan International Joint Laboratory of Animal Welfare and Health Breeding, College of Animal Science and Technology, Henan University of Science and Technology, Luoyang, China; 3 Innovative Research Team of Livestock Intelligent Breeding and Equipment, Science & Technology Innovation Center for Completed Set Equipment, Longmen Laboratory, Luoyang, China; 4 Faculty of Polytechnic Institute, Graduate School of Agricultural Sciences, The University of Shinshu, Matsumoto, Japan; 5 State Key Laboratory of Animal Nutrition, Department of Animal Nutrition and Feed Science, College of Animal Science and Technology, China Agricultural University, Beijing, China

**Keywords:** broiler health, HSD, inflammation, metabolic disorders, oxidative damage, stress

## Abstract

High stocking density (HSD) stress is a serious risk factor for poor animal health and loss of commercial productivity that affects broiler farms globally. While HSD can increase production efficiency and reduce costs, it can also lead to aggressive behavior and competition among the animals, unequal resource distribution, increased stress, disease, and loss of meat quality. Under HSD stress conditions, the redox balance is disrupted, inflammatory responses are exacerbated, immune function is impaired, and susceptibility to disease increases. This study reviewed the effects of HSD on the health of broilers, analyzing and prioritizing the associated physiological and biochemical changes. This review focuses on broiler chickens. HSD can have multiple adverse effects on poultry health, and this review evaluates nutritional strategies aimed at alleviating the negative effects of HSD stress to enhance welfare and productivity. By synthesizing current evidence, this review highlights the impacts of HSD on broiler health and welfare and identifies opportunities for nutritional interventions to mitigate these effects. This review provides a comprehensive reference for future research of HSD stress in poultry.

## 1 Introduction

Against the backdrop of global population growth and rising food demand, poultry farming has emerged as one of the most productive sources of protein worldwide (1). According to data from the United States Department of Agriculture (USDA), global broiler production reached 102,389,000 metric tons in 2023, an increase of 549,000 metric tons year-on-year, with accelerated growth anticipated in 2024. The modern poultry industry has undergone significant transformation, particularly with the widespread adoption of intensive farming systems, which have greatly increased production efficiency and output. In this context, China, as a leading player in the global poultry production market, has actively promoted large-scale broiler farming, particularly by means of the multi-tier cage system (2). However, despite the clear advantages of intensive systems in boosting production efficiency, the associated challenges of HSD have sparked considerable debate (3, 4).

Stocking density is defined as the number or total weight of birds per unit area (e.g., birds/m^2^ or kg/m^2^), and HSD has harmful effects on poultry physiology and behavior (5). These effects are influenced by genetic factors, flock size, environmental conditions (e.g., temperature, ventilation, lighting), and management practices (6). HSD is a multifactorial stressor, encompassing spatial constraints limiting mobility, respiratory distress, overheating, oxidative stress, increased human intervention from intensified management, psychological stress from social hierarchy disruption, immune suppression, metabolic dysregulation, and destructive behavior such as pecking (7–12). These factors can interact synergistically, creating a complex network of physiological and psychological stress. Chronic exposure to such stress not only impairs growth and feed conversion efficiency but also increases disease susceptibility.

To provide a more detailed description of how HSD is applied in experimental and commercial broiler production, Table 1 summarizes representative studies, detailing stocking density per unit area, housing system (e.g., floor pens in conventional or environmentally controlled houses, commercial broiler houses), exposure period, and the main reported effects of HSD on growth performance, health, and welfare.

**Table 1:** Representative studies on high stocking density (HSD) in broilers.

Stocking density	Housing system	Exposure period	Main reported impacts of HSD	References
5 → 20 birds/m^2^	Floor pens on litter, conventional broiler house, open/closed NR	Up to 42 days	Decreased final body weight and feed conversion ratio (FCR) at 42 d.	(23)
30 → 45 kg/m^2^	Floor pens on litter, environmentally controlled house, open/closed NR	Grow-out period	Reduced weight gain and feed intake; increased litter moisture, footpad lesions and skin scratches.	(24)
22 birds/m^2^	Ground litter pens, open/closed NR	28–42 days	Reduced production performance; intestinal mucosal damage; gut microbiota disruption; impaired digestion and absorption.	(25)
12, 15, 18 birds/m^2^ (commercial setting)	Commercial broiler house, likely floor system, open/closed NR	NR	Inhibited lymphocyte proliferation; increased mortality due to sudden death syndrome (SDS), especially at 18 birds/m^2^.	(29)

NR, not reported in the cited study.

Extensive research has identified HSD as the main constraint on modern poultry production, particularly during the critical growth period from mid-fattening to pre-slaughter (13, 14). Under HSD conditions, broilers are exposed to severe overcrowding and poor air quality, resulting in chronic stress (15). The primary mechanism involves hyperactivation of the hypothalamic–pituitary–adrenal (HPA) axis, leading to over-secretion of corticosterone, the primary avian stress hormone. This neuroendocrine imbalance systemically disrupts metabolic homeostasis and immune function, driving excessive production of the pro-inflammatory cytokines, TNF-*α*, IL-1β, IL-6, and exacerbating chronic inflammation (16). Prolonged stress can also induce vascular endothelial damage and intestinal barrier dysfunction, culminating in systemic inflammation and impaired growth performance (17). HSD disrupts redox homeostasis through multiple pathways, with overproduction of reactive oxygen species (ROS) serving as the primary cause of oxidative tissue damage (18). This loss of control is linked to the depletion or functional impairment of the antioxidant defense system involving superoxide dismutase, glutathione peroxidase, and vitamin E under persistent stress, which fail to neutralize excess ROS (19). The elevated oxidative stress accelerates lipid peroxidation, protein oxidation, and DNA damage, compounding the health risks to broilers under HSD.

Given the serious effects of HSD on poultry health, elucidating its mechanistic underpinnings is vital for developing effective mitigation strategies. In contrast to previous reviews, this article focuses specifically on broiler chickens reared under HSD, integrating organ-level evidence on oxidative damage, inflammation, and metabolic dysregulation with the evaluation of anti-inflammatory and antioxidant feed additives as targeted mitigation tools. This review comprehensively analyzed the impact of HSD stress on broiler chicken health, with the specific objectives of determining the pathways involved in oxidative stress, inflammatory cascades, and metabolic physiological and biochemical disturbances induced by HSD and evaluating various mitigation strategies to alleviate HSD-associated harm to broiler chickens.

## 2 Negative effects of HSD on broiler performance and health

To address the growing global demand for economically produced chicken meat, HSD has become a popular method to increase production efficiency and space utilization. However, intensive farming has also raised widespread concerns regarding animal welfare and healthful farming practices. The physiological regulation of growth and development of broilers is a multifactorial process, involving both genetic programming and environmental conditions, and overcrowding acts as a critical stressor that disrupts that process (20). HSD not only impairs growth performance and immune competence but also initiates cascading physiological stress responses, ultimately compromising poultry health through interconnected pathological mechanisms (21, 22). Studies indicate that when stocking density was increased from 5 birds/m^2^ to 20 birds/m^2^, the final body weight of broilers at 42 days of age significantly decreased, and the FCR was lower (23). Similarly, when SD based on broiler weight was increased from 30 kg/m^2^ to 45 kg/m^2^, broiler weight gain and feed consumption declined, while bedding moisture content and foot pad lesion scores increased linearly. At the same time, the number of scratches on the back and thighs of the birds also increased (24). These negative effects were particularly pronounced during the later fattening stages. Zhang et al. found that HSD (22 birds/m^2^) had a significant negative impact on the production performance of broilers during the rapid growth phase (28–42 days), with overcrowding leading to intestinal mucosal damage, gut microbiota disruption, and impaired digestion and absorption (25). In addition, HSD intensified abnormal behaviors such as feather-pecking and fighting for space, increased energy consumption and skin damage, and accelerated pathogen transmission, leading to higher incidence rates of *E. coli* infections, respiratory diseases, and cecal lesions (26–28). HSD (18 birds/m^2^) also inhibited lymphocyte proliferation and significantly increased the mortality rate from sudden death syndrome (SDS) (29). As homeothermic animals, broiler chickens need to balance their heat production and heat loss to maintain a stable body temperature (30). However, when the environmental temperature exceeds their comfort range, broilers exhibit a range of heat stress responses, such as increased water intake and reduced food consumption in an attempt at thermoregulation. The combined effects of high temperature and HSD lead to increased respiration rate and risk of respiratory tract damage (31, 32). In addition, overcrowding worsens air quality, resulting in higher concentrations of ammonia and carbon dioxide, which can irritate the respiratory tract and exacerbate disease risks (10). Long-term HSD farming can also cause medical problems such as tibial dyschondroplasia, with the incidence of these diseases increasing as stocking density rises (33).

Thus, as a complex chronic stressor, HSD disrupts broiler physiological homeostasis in several ways, including competition for space and resources, metabolic disorders such as reduced feed intake and increased water consumption, immune suppression, and unbalanced thermoregulation.

## 3 Stress mechanisms

Cannon first introduced the concept of stress in 1925 and proposed the “fight-or-flight” response as an immediate physiological reaction to threats (34). Building on this, Selye established a systematic stress theory in 1936 (35) and defined stress as “the nonspecific response of the body to any demand” (36). In this context, “nonspecific” denotes a shared constellation of physiological responses largely independent of stressor identity. Selye further described stress progression as the general adaptation syndrome (GAS), encompassing the alarm, resistance, and exhaustion stages (37).

Current theory holds that stress responses are governed by an integrated neuroendocrine network across multiple brain regions, with the amygdala as a key trigger (38). By rapidly detecting threat-related cues, the amygdala engages hypothalamic circuits to activate the sympathoadrenal medullary (SAM) system, eliciting a rapid emergency response (39). This response features adrenal-medullary release of adrenaline and noradrenaline, which act via *β*-adrenergic receptors to drive “fight-or-flight” adaptations—elevated heart rate and blood pressure, accelerated respiration, and rapid mobilization of energy substrates (40). With prolonged or chronic stress, activation shifts toward the hypothalamic–pituitary–adrenal (HPA) axis: ACTH stimulates the adrenal cortex to produce glucocorticoids, primarily corticosterone (CORT) in poultry (41, 42). In HSD environments, physical crowding and increased agonistic interactions elevate sympathetic outflow, leading to repeated surges of catecholamines. Such sustained adrenergic activation differs from acute stress models and contributes to chronic metabolic strain and heightened oxidative load in broilers raised at high density.

High stocking density is a key promoter of HPA-mediated stress responses in poultry. In environments with limited space for movement, poultry often exhibit abnormal changes in endocrine indicators and altered behavioral patterns. Dai et al. (16) confirmed that when broilers were exposed to HSD environments, the HPA axis was activated, leading to a significant increase in serum CORT levels, accompanied by elevated release of lipopolysaccharide (LPS), IL-1β, and TNF-*α*, resulting in increased pathological damage and decreased production performance. Therefore, serum CORT levels can serve as a reliable biomarker of stress in poultry (43). HSD has been consistently shown to induce tonic activation of the HPA axis, with elevated baseline corticosterone reflecting persistent environmental pressure rather than episodic threat detection. This chronic glucocorticoid exposure underlies many of the metabolic, immune, and behavioral alterations described in broilers subjected to high stocking density.

**Figure 1:**
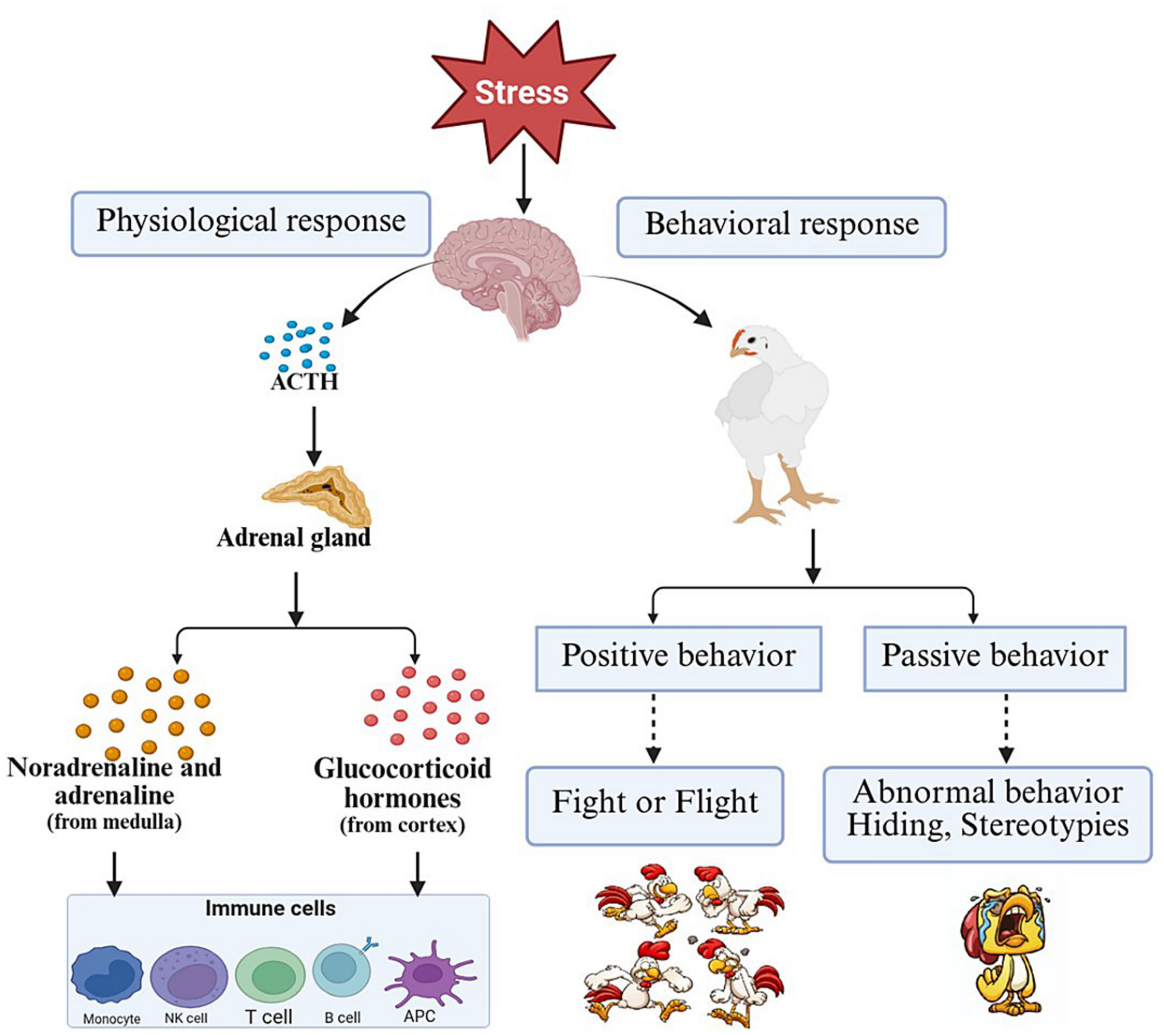
The impact of stress on animal physiology and behavior.

As a central hub for higher cognitive regulation, the prefrontal cortex assesses the nature and degree of threat posed by stressors and modulates emotional responses via dopaminergic pathways from the pregenual cingulate to the ventral tegmental area. The hippocampus, through a glucocorticoid receptor-mediated negative feedback mechanism, inhibits excessive activation of the HPA axis, restoring homeostasis (44). Under HSD, continuous exposure to crowding-associated stressors impairs these regulatory circuits, reducing the efficiency of hippocampal negative feedback on the HPA axis and thereby intensifying corticosterone-driven physiological dysregulation. It is important to note that stress not only includes physiological stress (such as inflammation and pain) but also psychological stress (such as depression and fear) (45). Studies have shown that with increased stocking density, animal fear behaviors and the incidence of footpad dermatitis increase (46). Behavioral evidence further supports the psychological burden of HSD, as fearfulness, agitation, and abnormal social interactions increase markedly with rising density, indicating that both physical and psychological components of stress coexist and potentiate one another under overcrowded conditions. While the stress response itself is a protective mechanism for the body to adapt to the environment, prolonged stress can lead to a variety of adaptive diseases (47). As illustrated in Figure 1, HSD acts as a persistent environmental stressor that concurrently triggers neuroendocrine, metabolic, immune, and behavioral pathways. The figure highlights how spatial restriction, social conflict, and impaired air quality collectively activate the amygdala–hypothalamus circuits, leading to sustained stimulation of both the SAM and HPA axes. This HSD-driven activation propagates downstream effects including altered energy allocation, inflammatory sensitization, oxidative imbalance, and suppressed immune function, forming the mechanistic basis for the physiological disturbances observed in high-density broiler systems. Through these complex neuroendocrine mechanisms, the brain effectively perceives, integrates, and regulates changes in both internal and external environments, aiding the body to adapt to various stress stimuli and maintain physiological and psychological balance.

## 4 The influence of HSD on the main organs of poultry

### 4.1 Brain

Although the avian brain is relatively small, it achieves highly complex physiological regulation through specialized modular structures (48). As the central hub of neuroendocrine integration, the hypothalamus precisely regulates metabolic homeostasis through the neuropeptide network of the arcuate nucleus. Because feeding is essential for animals to maintain metabolism and growth, the hypothalamus plays a key role in body weight regulation, feeding behavior, and energy balance (49).

In regard to the feeding behavior of birds, the hypothalamus contains various neuropeptides involved in appetite regulation, which can be categorized based on their effects as appetite-promoting or appetite-suppressing neuropeptides (50). Appetite-promoting neuropeptides include neuropeptide Y (NPY), agouti-related peptide (AGRP), and orexin, while appetite-suppressing neuropeptides include pro-opiomelanocortin (POMC) and its derivative *α*-melanocyte-stimulating hormone (α-MSH) (51). The arcuate nucleus of the hypothalamus is a key region for regulating feeding behavior. Neuropeptides such as NPY and AGRP are expressed in neurons within this region and modulate feeding behavior by projecting to secondary neurons in areas such as the paraventricular nucleus, lateral hypothalamus, and periventricular zone. Secondary neurons located in the lateral hypothalamus primarily express orexin and melanin-concentrating hormone (MCH), both of which promote feeding (52, 53). In addition to NPY/AGRP, POMC and cocaine- and amphetamine-regulated transcript (CART) are also expressed in arcuate nucleus neurons. Unlike NPY/AGRP, POMC and CART are expressed in different neurons within the arcuate nucleus. POMC/CART primary neurons can also project to secondary neurons located in the paraventricular nucleus, lateral hypothalamus, and periventricular zone of the hypothalamus (54).

Studies have found that under HSD conditions, the expression levels of NPY and AGRP in broilers are closely related to changes in food intake, with NPY expression reduced and AGRP showing a downward trend, while POMC gene expression decreases, in opposition to its appetite-suppressing effects (55). In addition, under restraint stress, both body weight and food intake in mice significantly decreased along with the ghrelin mRNA level in the hypothalamus, while the POMC mRNA level significantly increased (56). Ghrelin is an appetite-promoting factor, while POMC, through its product *α*-MSH, inhibits appetite and activates melanocortin receptors to suppress feeding. These results suggest that broilers in high-density rearing environments may face stronger competition for resources or the environment, which further exacerbates the occurrence of stress responses.

In growth regulation, the hypothalamus-pituitary-growth axis promotes the secretion of growth hormone (GH) through growth hormone-releasing hormone (GHRH), which is strictly regulated by somatostatin (SST). GH further induces the synthesis of insulin-like growth factor 1 (IGF-1) in the liver through growth hormone receptors (GHR), thereby promoting the development of bones and muscles (57). Numerous studies have shown that high-density rearing increases inflammation in broilers, and the release of pro-inflammatory factors like TNF-*α*, IL-1β further triggers leptin resistance, weakens AGRP neuron function, and induces metabolic reprogramming, ultimately leading to reduced food intake, nutritional imbalance, and stunted growth (58, 59). In addition, chronic stress activates the HPA axis, resulting in sustained elevation of glucocorticoids, which suppresses the GH/IGF-1 signaling pathway and reduces GHRH sensitivity (60). This structural-functional cascade reveals the systemic impact of high-density rearing on poultry production performance through the hypothalamus-mediated neuroendocrine network.

### 4.2 Liver

The liver is not only the largest digestive gland in an animal’s body, but also the core organ for redox regulation, energy metabolism conversion, and the synthesis of various substances in the body for maintaining physiological homeostasis (61). The liver receives dual blood supply from the systemic circulation (hepatic artery) and digestive system (portal vein), executing critical functions including nutrient metabolism, xenobiotic detoxification, and immunoregulation (62). The portal venous system serves not only as a conduit for nutrient transport but also delivers gut-derived exogenous substances, digestive metabolites, senescent erythrocytes, and microbial components with immunogenic potential (63).

The central nervous system (CNS) regulates hepatic immunity under stress through two primary pathways. First, the HPA axis releases glucocorticoids, which bind to hepatic glucocorticoid receptor alpha (GRα), influencing glucose metabolism and inflammation (64). Second, the sympathetic nervous system (SNS) is activated via splanchnic nerves, releasing norepinephrine that activates β2-adrenergic receptors on hepatocytes and Kupffer cells (65). These mechanisms collectively modulate the liver’s immuno-metabolic responses during stress.

Oxidative stress and inflammation are closely related in a variety of pathological processes; they occur simultaneously and promote each other, especially at damage sites (66). Studies have shown that HSD induces oxidative damage in the liver and triggers chronic inflammation by increasing reactive oxygen species (ROS) in broilers (67). A similar phenomenon has also been reported in laying ducks, where HSD significantly weakened their antioxidant capacity, thereby affecting egg production performance and egg quality (68). Similar hepatic lesions and oxidative-stress signatures have been described in rodent restraint-stress models, which helps contextualize stress-related inflammatory and redox pathways in the liver (69, 70).

In fish, density stress similarly disrupted the dynamic balance of energy reserves and expenditure, requiring the body to reallocate metabolic resources to meet the increased energy demands (71). Similarly, HSD can cause lipid metabolic disorders in poultry by affecting lipid synthesis, degradation, and transport. Excessive lipid accumulation can cause liver toxicity, leading to liver dysfunction and inflammation. This sustained metabolic load disrupts the balance between lipid synthesis and breakdown in liver cells, ultimately leading to pathological fat deposition (72, 73).

Mitochondria play a crucial role in cellular energy production through oxidative phosphorylation, which supports essential biological processes (74, 75). External stimuli can inhibit the replication of mitochondrial DNA, reduce mitochondrial biogenesis, and ultimately mitochondrial numbers (76). HSD environments increase markers of oxidative damage in liver mitochondria, such as elevated MDA levels, reduced GSH content, and decreased ATP levels. These changes affect mtDNA replication and significantly disrupt mitochondrial morphology and function in the livers of broilers (77). These results confirm the negative impact of HSD on liver energy metabolism.

The liver has traditionally been considered a sterile organ, but recent studies have challenged this view by demonstrating the presence of microorganisms in the livers of various animals (78). Research in cattle, dogs, and mice has identified bacterial communities within the liver parenchyma, suggesting that the liver may possess its own microbiome (79–81). Another study found an increased abundance of *Pseudomonas aeruginosa*, particularly associated with weakened hepatic antioxidant capacity, in the livers of broilers raised under HSD (67). This finding suggests a potential link between the hepatic microbiome and antioxidant defense. *P. aeruginosa* has been reported to induce oxidative stress in host cells by producing ROS through virulence factors (82, 83). Across poultry species, HSD is consistently linked to oxidative/metabolic disturbance, but the dominant physiological trade-offs appear to differ. In broilers and laying ducks, reduced antioxidant capacity aligns with impaired production traits, whereas geese show elevated citrate and L-malate under HSD, suggesting a stronger shift in TCA-cycle–related energy metabolism.

### 4.3 Intestine

The avian intestinal system is a highly specialized and efficient tubular structure for digestion. Based on the order of food passage, it can be divided into the crop, proventriculus, gizzard, small intestine, large intestine, and cloaca (84). The crop serves as a temporary storage organ, where mucus is secreted to soften the ingesta. In some species, such as pigeons, it can also secrete crop milk to feed the young. The food then enters the proventriculus for chemical digestion, where strong acidic gastric juices and proteases initiate protein breakdown. The gizzard, with its thick muscular layer and grit, mechanically grinds hard food particles within 2–4 h, while a keratinized lining prevents tissue damage. The small intestine (comprising the duodenum, jejunum, and ileum) completes nutrient breakdown with the help of pancreatic enzymes and bile, while densely packed villi efficiently absorb nutrients like monosaccharides and amino acids. The paired ceca in the large intestine ferment cellulose through microbial action, while the rectum concentrates feces. Ultimately, all metabolic waste is expelled through the cloaca, which consists of three chambers (fecal, urinary, and rectal passages) (85). This short yet efficient intestinal structure, along with a rapid transit rate, meets the high metabolic demands of birds.

When food enters the small intestine, fine, microscopic intestinal villi on the surface of the intestinal mucosal epithelial cells increase the absorptive surface area, enhancing nutrient absorption efficiency, while also protecting the intestinal mucosa from damage and irritation. Villus height (VH), crypt depth (CD), and the ratio of villus height to crypt depth (VCR) are key indicators of intestinal morphological structure. VH represents the number and absorptive area of mature intestinal villus cells, while CD indicates the maturity of crypt cells; thus, the shape of the intestinal villi can influence the growth and development of the organism. Research has found that HSD at 21 days of age reduced the VH, the VCR, and the villus surface area in the duodenum, while increasing the number of intraepithelial lymphocytes (IELs) in the ileum (86). The impact of HSD on morphological parameters was even more pronounced at 42 days, affecting the villus surface area across all segments of the small intestine. These findings are consistent with the study by Kridtayopas et al. (87), which reported a decrease in villus height in broiler chickens due to HSD. Kamel et al. (88) also observed negative effects of HSD on the morphology of duodenal tissue. At the same time, studies have found that the feed conversion ratio (FCR) in broiler chickens was positively correlated with the VCR. A decrease in villus height is accompanied by a significant decline in FCR (89). In addition, villus damage leads to a decrease in disaccharidase activity, and undigested lactose ferments at the rear end of the intestine, causing osmotic diarrhea (90).

Tight junction proteins, including claudins, occludin, and adhesion molecules, are structures located between the epithelial cells of the intestinal wall that regulate paracellular permeability and the exchange of substances between cells, serving as important components of the intestinal epithelial barrier (91). These tight junction proteins form a robust barrier that prevents bacteria, toxins, and large molecules from indiscriminately entering the intestinal tissue while allowing water, nutrients, and certain small molecules to pass through (92). An increase in stocking density leads to broiler chickens being in a prolonged state of HSD stress, which in turn causes persistent activation of the HPA axis, resulting in elevated serum corticosterone levels. This suppresses the mRNA transcription of claudin-1, occludin, and ZO-1, while accelerating the degradation of tight junction proteins through the ubiquitin-proteasome system, and impairing the binding of ZO-1 to the cytoskeleton (25). Consequently, the intestinal barrier structure is compromised, leading to increased intestinal permeability and chronic intestinal inflammation. Under stress conditions, the production of ROS in the intestine exceeds the body’s regulatory capacity, causing an imbalance in redox homeostasis and leading to the oxidation of thiol groups in claudin and occludin, which results in a loss of activity (93).

The occurrence of intestinal barrier damage is often accompanied by dysbiosis of the gut microbiota (94). The primary function of gut microorganisms is to provide nutrients to the intestinal epithelial cells and the host through their metabolic products, enhance host immunity, help the host resist foreign invasions, and perform various functions (95, 96). For example, gut microbiota can not only influence the formation of intestinal blood vessels but also provide essential short-chain fatty acids (SCFAs) and vitamins, as well as assist in the digestion of dietary fiber (97). However, HSD not only affects the intestinal morphology of broiler chickens but also alters the composition of the gut microbiota. *Lactobacillus* has been confirmed as a beneficial bacterium in the intestine, competitively hindering the colonization of pathogenic bacteria in the gut, promoting the development of intestinal villi, and enhancing nutrient absorption (98). It plays an important role in maintaining the functional integrity of the intestinal microbial barrier. Studies have found that when the stocking density reaches 39 kg/m^2^, the population abundance of Lactobacillus is significantly reduced, indicating a decline in this beneficial bacterial group under HSD conditions (99). In a study on the gut microbiota of ducks at different stocking densities, it was found that excessive HSD significantly increased the relative abundance of *Firmicutes* in the gut, while the ratio of Firmicutes to Bacteroidetes, which is associated with energy metabolism, is also significantly elevated (100). Research has found that in the intestines of broiler chickens under HSD stress, the population levels of Faecalibacterium increase, whereas those of Lactobacillus and Bifidobacterium decrease, reflecting shifts in key microbial populations. These changes collectively contribute to dysbiosis of the intestinal microbiota, which may impair nutrient utilization and reduce production performance (101, 102). Disruption of the structure of the gut microbiota in poultry can reduce the efficiency of nutrient absorption and decrease feed utilization, thereby adversely affecting poultry production performance (103). Across poultry, higher stocking density is consistently associated with gut microbiota disruption, though the specific signatures vary by species. Broilers commonly show reduced beneficial taxa (e.g., *Lactobacillus*, *Bifidobacterium*) with enrichment of potentially pro-inflammatory microbes, ducks tend to exhibit an increased Firmicutes/Bacteroidetes ratio, and geese show altered cecal fermentation metabolites consistent with shifts in fiber and lipid utilization.

Overall, the brain, liver and intestine emerge as major target organs of HSD in poultry. Hypothalamic dysregulation alters feed intake and growth hormone signaling, hepatic oxidative injury and lipid dysmetabolism compromise nutrient handling, and intestinal barrier disruption together with dysbiosis undermines digestion and immune defense. By integrating these organ-level responses, HSD can be understood as a systemic condition in which central and peripheral dysfunctions.

## 5 The mechanism of oxidative damage occurrence

During the growth of broiler chickens, HSD stress can disrupt the body’s redox homeostasis through various mechanisms, with excessive generation of ROS leading to oxidative tissue damage (104). ROS includes superoxide anion (O₂−), hydrogen peroxide (H₂O₂), and hydroxyl radicals (OH−). When these highly reactive oxygen metabolites accumulate excessively in the body, they can cause lipid peroxidation, oxidative modification of proteins, and DNA damage through chain reactions, with severe damage to tissue structure and function (105).

Under stress conditions, the production of ROS primarily relies on enzymatic reactions mediated by the membrane-bound NADPH oxidase family (NOXs) and the generation of O₂^−^ due to electron leakage at the end of the mitochondrial electron transport chain (106). The NOX family consists of seven members, including NOX1 to NOX5, as well as DUOX1 and DUOX2. All of the NOX family proteins are homologous, containing a conserved NADPH oxidase functional domain in their core structure; however, the DUOX subtypes have additional calcium-binding regions and peroxidase-like domains (107). These members are distributed across various subcellular structures, such as the plasma membrane, endoplasmic reticulum, and mitochondria, participating in various physiological and pathological processes by catalyzing the local generation of ROS (108). Research has shown that acute heat stress can rapidly increase the expression of NOX4 in the liver of broiler chickens within 1 h, with a simultaneous rise in the expression of superoxide dismutase (SOD) and other enzymes that mitigate oxidative stress (109).

During the process of oxidative phosphorylation, some unused electrons may undergo non-enzymatic reactions with oxygen, leading to the generation of ROS: single-electron leakage producing O₂^−^ and double-electron leakage producing H₂O₂. Under basal metabolic conditions, approximately 2–4% of the electrons transferred to the electron transport chain leak prematurely, resulting in the production of superoxide (110, 111). HSD inhibits the activity of mitochondrial respiratory complexes I and III in broiler chickens, leading to dysfunction of the electron transport chain and impaired ATP synthesis, which in turn triggers electron leakage and significantly increases ROS levels (77). In addition to NOXs and the electron transport chain, H₂O₂ can also be produced by oxidases and peroxisomes in the endoplasmic reticulum and other subcellular structures. Lipid oxidation is also an important source of ROS. Lipid hydroperoxides and their radicals (peroxyl radicals and alkoxy radicals), generated from the oxidation of polyunsaturated fatty acids, not only contribute to oxidative damage but also regulate redox signaling and the immune response (112). For example, lipoxygenases and cyclooxygenases produce reactive oxidizing intermediates that directly regulate the activation of inflammatory responses (113).

Under normal physiological conditions, the body relies on both enzymatic antioxidant systems, superoxide dismutase (SOD), catalase (CAT), and glutathione peroxidase (GSH-Px), and non-enzymatic systems, glutathione (GSH), vitamin C, and vitamin E to eliminate free radicals, and maintain redox balance (114). However, loss of oxidative homeostasis from HSD leads to accumulation of free radicals, lipid peroxidation reactions in the cell membrane, and the production of toxic aldehyde products such as malondialdehyde (MDA) and 4-hydroxynonenal (4-HNE) (115). These substances exacerbate cellular dysfunction by inducing protein cross-linking, denaturation and DNA damage. Research by Cai et al. (116) has shown that HSD reduces total antioxidant capacity (T-AOC) in broiler chickens with decreased SOD and CAT activity and increased MDA levels.

Under HSD, the serum T-AOC in broiler chickens significantly decreases, while protein carbonylation (PCO) product levels increase, concomitant with systemic oxidative damage. Accumulation of 4-HNE induces the PCO reaction by covalently modifying cysteine residues, and the PCO products generated from this reaction can serve as biomarkers for assessing the degree of oxidative damage (117). Sustained oxidative stress may also dysregulate the HPA axis and increase CORT, which exacerbates oxidative and neuroendocrine disturbances and may culminate in DNA damage, apoptosis, and impaired growth (118, 119).

In conclusion, oxidative damage under HSD arises from the convergence of enhanced ROS generation and weakened antioxidant defenses. Up-regulated NOX activity, electron leakage from dysfunctional mitochondrial complexes and lipid peroxidation jointly drive the accumulation of toxic aldehydes and protein carbonyls, while endogenous enzymatic and non-enzymatic antioxidant systems become progressively exhausted. This redox imbalance not only damages cellular macromolecules but also affect neuroendocrine and inflammatory pathways, thereby magnifying the systemic impact of density stress.

## 6 Inflammatory overactivation

Inflammation is an adaptive defense mechanism in multicellular organisms that evolved over a long period of evolutionary time (120). When a body encounters pathogenic invasion or cellular damage, it rapidly initiates a program involving the innate immune system and precisely regulated vascular and enzymatic processes to fend off the attack and repair tissue damage (121). This response mobilizes an army of dendritic cells, macrophages, neutrophils, and epithelial cells, which recognize microbe-associated molecular patterns (MAMPs) and damage-associated molecular patterns (DAMPs) through pattern recognition receptors (PRRs) to mount an effective inflammatory response (122). The PRR family includes transmembrane Toll-like receptors (TLRs) and intracellular NOD-like receptors (NLRs), which recognize specific pathogen molecular patterns (123). For example, TLR4 recognizes lipopolysaccharides from Gram-negative bacteria, while TLR9 recognizes microbial DNA (124, 125). TLR recognition activates key signaling pathways, such as NF-κB and mitogen-activated protein kinase (MAPK), inducing the expression of pro-inflammatory mediators and antimicrobial peptides to protect the body from infection (126). However, in broiler production, HSD may lead to overactivation of the immune system and chronic inflammation (127). Research by Dai et al. (16) has confirmed that HSD stress damages the intestinal barrier in broilers and increases serum LPS. LPS activates the TLR4/NF-κB signaling pathway, thereby promoting the transcriptional expression of pro-inflammatory cytokines, including IL-1β and TNF-*α* transcription, which exacerbates intestinal inflammation and reduces broiler growth performance. Zhao et al. (55) showed that serum levels of TNF-α and IL-1β were significantly elevated in broilers under HSD stress, and this effect was most likely related to activation of the hypothalamic MAPK signaling pathway.

The NF-κB and MAPK signaling pathways drive the expression of inflammatory mediators and enzymes involved in the inflammatory response (128). When exposed to external stimuli, the NF-κB pathway phosphorylates the inhibitor of kappa B alpha (IκBα), allowing NF-κB to enter the nucleus and activate downstream effector molecules in the MAPK pathway, which together promote the expression of inflammatory cytokines, such as cyclooxygenase-2 (COX-2). COX-2 plays an important role in inflammation by converting arachidonic acid into prostaglandin H2 (PGH2), which is then further converted into prostaglandin E2 (PGE2) under the action of membrane prostaglandin E synthase-1 (129). PGE2 binds to the prostaglandin E receptor family (EP1-EP4), triggering various signaling pathways that regulate the inflammatory response, immune response, and local blood flow. This can induce an array of physiological activities, including stress responses, cell proliferation, and cell death (130). Previous studies have confirmed that EP4 receptor activation mediates inflammation and neuropathic pain (131). In a restraint stress rat model, high levels of PGE2 in damaged tissues activated co-expression of EP4 and the capsaicin receptor in the dorsal root ganglion, mediating pain sensitization, while EP4 antagonists effectively alleviated the inflammatory response and neuropathic pain (132). Although this work was conducted in rats, the TLR4–NF-κB–COX-2–PGE₂–EP4 axis is highly conserved and likely contributes to HSD-associated inflammatory and nociceptive signaling in poultry as well. Research by Liu et al. (133) revealed that LPS stimulation activated IL-1β release through the MAPK/NF-κB signaling pathway, subsequently upregulating the expression of COX-2, mPGES-1, and EP4, which inhibited broiler growth.

## 7 Mechanisms of energy metabolism regulation in avians

Energy metabolism is a core process that maintains the physiological functions of organisms (134). Cells generate ATP by breaking down glucose, fats, and amino acids through the respiratory pathways, a process that plays a crucial role in both physiological and pathological states (135). These metabolic pathways work together through key steps such as glycolysis, the tricarboxylic acid (TCA) cycle and oxidative phosphorylation and are precisely regulated and controlled by enzymes and metabolic products. In order to maintain their high body temperature and metabolic rate, birds must have a very efficient energy supply system (136). Under stress conditions, the activation of the HPA axis produces elevated serum CORT levels, which causes an imbalance in nutrient allocation, thereby disrupting the coordinated operation of metabolic pathways (137).

### 7.1 Glycolysis

Glycolysis is the metabolic process through which glucose is broken down into pyruvate or lactate via enzyme-catalyzed reactions, generating ATP (138). As one of the fundamental energy pathways, glycolysis can maintain cell function under hypoxic conditions by rapidly producing energy (139). During inflammatory responses, immune cells upregulate glycolytic activity to meet their high energy demands, and the enhanced glycolytic metabolism can amplify the inflammatory cascade by increasing the synthesis of pro-inflammatory cytokines and ROS (140). Under acute heat stress conditions, significant disturbances occur in the metabolic and endocrine systems of broilers, characterized by accelerated plasma protein breakdown, increased glucose levels, and decreased circulating levels of triiodothyronine (T3) (141). These changes are closely related to the activation of inflammatory responses and glycolytic pathways.

Long-term exposure of poultry to stress can affect their muscle metabolism. For example, heat stress accelerates muscle glycolysis, leading to a rapid decrease in pH and reduced water-holding capacity of the muscles, thereby increasing the risk of pale, soft, exudative (PSE) meat (142). Research by Wu et al. (143) found that, compared to a low-density group, broilers in a HSD group exhibited significantly higher cooking loss rates, greater decreases in pH, and increased lactate dehydrogenase activity in their breast muscles. Transcriptomic analyses further indicated that under HSD conditions, the expression of genes related to protein hydrolysis, glycolysis, and immune stress was upregulated in the muscles of broilers, while the expression of genes associated with muscle growth, cell adhesion, and collagen synthesis was downregulated (144). The study by Ebeid et al. (145) confirmed that HSD significantly reduced the sensory quality scores of broiler meat, suggesting that abnormal activation of glycolysis under HSD may be the primary cause of lower meat quality in broilers.

### 7.2 TCA cycle

The TCA cycle is an intermediate pathway in the metabolism of carbohydrates, fats, and amino acids (146). This cycle begins with the oxidative decarboxylation of pyruvate, a product of glycolysis, to form acetyl-CoA, which then reacts with oxaloacetate to produce citrate (147). During this cycle, citrate is isomerized to isocitrate, then undergoes two oxidative decarboxylation reactions to generate *α*-ketoglutarate and succinyl-CoA, accompanied by substrate-level phosphorylation to produce one molecule of guanosine triphosphate (GTP) (148). Ultimately, succinyl-CoA completes the cycle by regenerating oxaloacetate. Throughout this process, the TCA cycle generates three molecules of nicotinamide adenine dinucleotide (NADH), one molecule of flavin adenine dinucleotide (FADH2), and one molecule of GTP, which provide a significant amount of reducing equivalents for ATP synthesis through the mitochondrial respiratory chain (149).

Under stress conditions, changes in metabolite levels in poultry can disrupt the TCA cycle, thereby affecting metabolic balance (150). For instance, under heat stress in broilers, the levels of key intermediate metabolites in the TCA cycle (such as L-malate and citrate) and the microbial metabolic product isobutyrate in the cecum are significantly reduced, indicating that TCA cycle activity is suppressed, which may lead to insufficient energy and impaired intestinal barrier function (151). In contrast, in geese under HSD stress environments, the concentrations of citrate and L-malate increase, while ribonucleic acid levels decrease, indicating enhanced TCA cycle activity. These metabolites may be converted into glucose through the TCA cycle to meet high energy demands but may also trigger inflammatory responses due to the accumulation of pro-inflammatory substrates (152). Therefore, the regulation of the TCA cycle under stress has a dual nature. Thus, although broilers, ducks and geese all exhibit TCA-cycle perturbations under density-related stress, the direction and extent of metabolite changes differ by species, suggesting that quantitative thresholds for HSD and optimal mitigation strategies may need to be tailored to each poultry species rather than extrapolated directly from Gallus-based data.

### 7.3 Oxidative phosphorylation

The mitochondrial oxidative phosphorylation system is a core component of cellular metabolism (153). The protein complexes, I-IV, coenzyme Q, and cytochrome c distributed on the mitochondrial inner membrane cristae must assemble into super-complexes to maintain normal function. Together with complex V (F1F0-ATP synthase), they complete the oxidative phosphorylation apparatus for ATP production, which is the primary energy carrier in nearly all cellular processes (154).

The mitochondrial membrane potential, driven by the formation of a proton gradient, is a critical factor for ATP synthesis, its decline typically indicating damage to the oxidative phosphorylation system (155). Evidence shows that HSD significantly reduces the mitochondrial membrane potential in the liver of broilers and inhibits the activity of Na^+^/K^+^-ATPase and Ca^2+^/Mg^2+^-ATPase. These changes inhibit ATP synthesis by disrupting ion gradients and reducing oxidative phosphorylation efficiency (102). Furthermore, the activity of the electron transport chain complexes (I-IV) directly influences oxidative phosphorylation efficiency, and their reduced activity typically impedes normal electron transfer, resulting in increased superoxide formation (156). Yang et al. (77) found that, compared to a low-density group, broilers in the HSD group exhibited decreased activity of complexes I and III in liver tissue, accompanied by elevated levels of MDA, and reduced levels of glutathione (GSH) and ATP. This confirms the importance of complexes I and III in oxidative stress, which increases superoxide leakage, adversely affecting cellular energy metabolism and redox status. Thus, HSD has a significant impact on mitochondrial oxidative phosphorylation and overall cellular metabolism.

**Figure 2:**
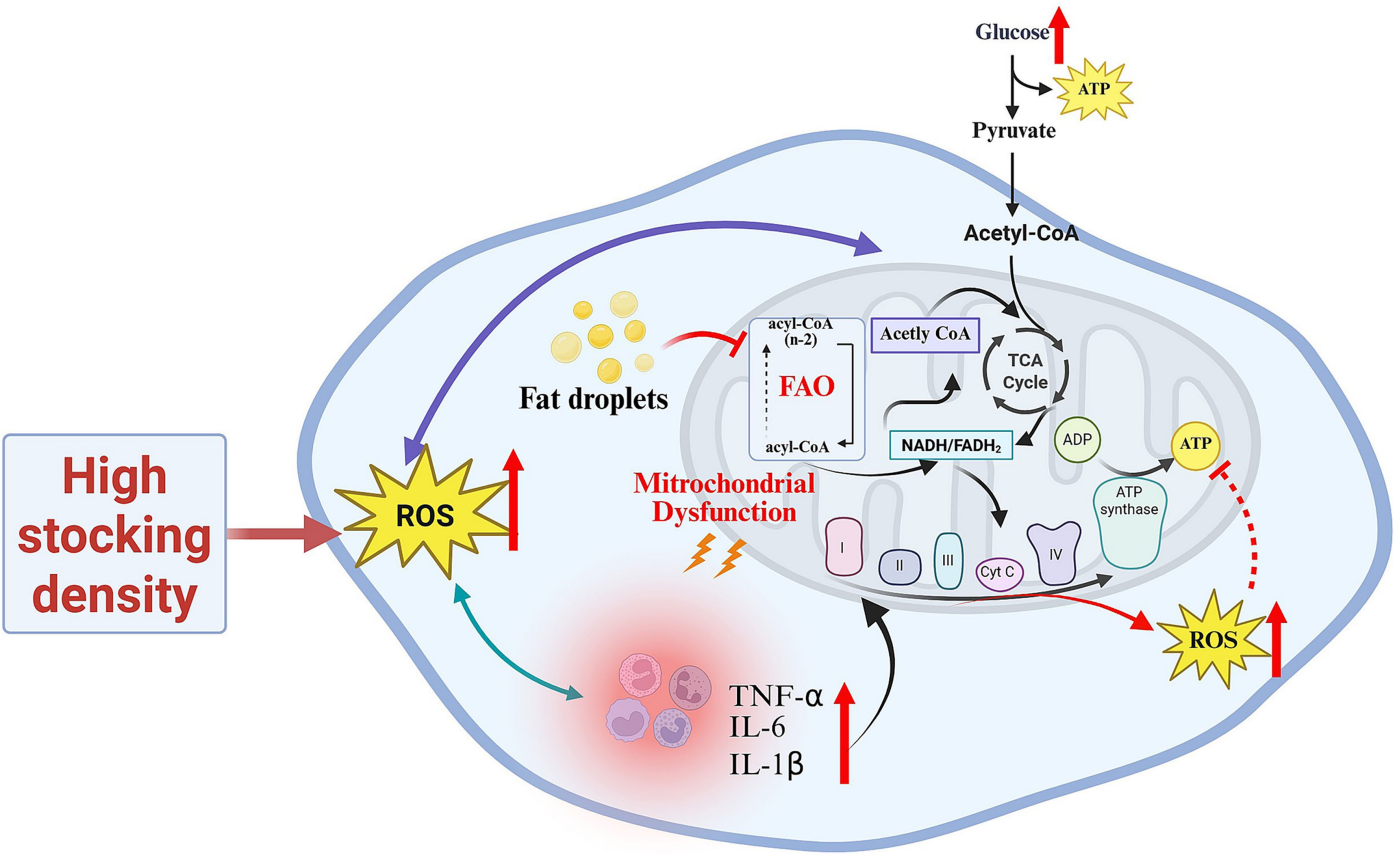
The effects of high-density stress on energy metabolism in poultry.

Figure 2 summarizes the metabolic consequences of HSD by integrating glycolysis, the TCA cycle, and mitochondrial oxidative phosphorylation into a unified model. HSD-induced activation of the HPA axis elevates corticosterone, which redirects nutrient allocation toward stress adaptation and enhances glycolytic flux. Concurrently, impaired TCA cycle turnover and reduced activities of electron transport chain complexes I and III compromise mitochondrial ATP generation, increasing ROS leakage and amplifying oxidative damage. By visually linking these pathways, Figure 2 clarifies how HSD disrupts energy metabolism at multiple regulatory nodes.

## 8 Mitigating HSD stress in poultry via anti-inflammatory and antioxidant feed additives

As the heart of intensive poultry production, HSD can significantly improve production efficiency, but overcrowding causes systemic damage, metabolic disorders, immune suppression and oxidative stress. Studies have demonstrated that HSD stress triggers ROS/inflammatory factor cascades through dual mechanisms of HPA axis activation and mitochondrial ETC dysfunction. This pathophysiological process disrupts the body’s redox homeostasis while compromising intestinal barrier integrity, resulting in diminished production performance. In this chapter, the mechanisms and application of chlorogenic acid (CGA), vitamin E and selenomethionine (SeMet) for alleviating the negative effects of HSD are reviewed.

### 8.1 Chlorogenic acid (CGA)

Chlorogenic acid (CGA) is a bioactive dietary polyphenol, widely distributed in medicinal plants such as honeysuckle (*Lonicera japonica*) and Eucommia (*Eucommia ulmoides*). Its biosynthesis involves the shikimate pathway (157). Because of its multiple functions, including antibacterial, anti-inflammatory, and heat-clearing properties, CGA has shown broad potential applications in medicine, food, healthcare, and organic syntheses. In recent years, CGA has been referred to as “plant gold,” and its use as a feed additive for animal health has attracted considerable attention, especially in response to the reduction or ban of antibiotic usage in agriculture. The pharmacokinetics of CGA have been elucidated in many different species. In rat models, Lafay et al. (158) confirmed that a small amount of the parent compound is directly absorbed in the stomach, while most CGA is hydrolyzed by esterases into caffeic acid and quinic acid once it reaches the intestines (159). These metabolites are then transported into the bloodstream through intestinal epithelial cells. Plasma primarily contains metabolites such as hydroxycinnamic acid derivatives, rather than CGA itself (160).

In HSD poultry farms, the addition of CGA to feed significantly improved the height of the ileal villi and the villus height-to-crypt depth ratio in broiler chickens. It also restored the expression of tight junction proteins (OCLN and ZO-1), reduced the levels of TNF-*α*, IL-1β, and IL-6, and decreased the abundance of harmful bacteria in the gut (161). CGA also mitigated oxidative stress by restoring SOD/GSH-Px activity, enhanced intestinal barrier function by upregulating tight junction protein expression, and restored balance of cecal microbiota by enriching beneficial bacteria such as *Blautia* (14). CGA also significantly lowered oxidative stress, which improved the meat quality of HSD broiler chickens. By activating the Nrf2 pathway, CGA significantly improved meat quality under oxidative stress, by restoring normal muscle pH, water-holding capacity, and color. Research has shown that the antioxidant effects of CGA are closely related to its regulation of metabolites. Four potential biomarkers—pyrimidine/purine metabolism, propionate metabolism, phenylalanine metabolism, and lysine metabolism—have provided new directions for future research on meat quality assessment (162).

In studies on the effects of heat stress on chicken embryos, eggs were injected with different doses of CGA into the amniotic cavity of chicken embryos, followed by heat stress treatment. Eggs injected with 4 mg of CGA exhibited significant antioxidant benefits, with a marked reduction in MDA levels, and a significant increase in the activity of superoxide dismutase (SOD), glutathione peroxidase (GSH-Px), and catalase (CAT) (163). Moreover, CGA significantly increased intestinal microbiota diversity, promoted the production of short-chain fatty acids, and enhanced the expression of the immune-related proteins, thymosin *β* and legumain, by activating PPAR and MAPK signaling pathways. It also elevated the levels of health-promoting metabolites such as 2,4-dihydroxybenzoic acid, providing new insights into the application of CGA in improving immune function and gut health in broiler chickens (164).

### 8.2 Vitamin E

In recent years, the application of vitamins as feed additives has gained increasing attention. Vitamin E, comprising tocopherols and tocotrienols, is a fat-soluble nutrient with potent antioxidant properties (165). It protects cellular membranes and tissues from free radical-induced lipid peroxidation while modulating enzyme activity (166). Among its isoforms, *α*-tocopherol, the most biologically active form, participates in the glutathione peroxidase pathway to combat oxidative damage. Due to its lipid solubility, vitamin E serves as an ideal membrane-bound antioxidant. Poultry require dietary vitamin E supplementation as they cannot synthesize it endogenously. Under heat stress, elevated levels of corticosterone and catecholamines induce lipid peroxidation in cellular membranes. Studies demonstrate that vitamin E enhances the survival, proliferation, and functionality of lymphocytes, macrophages, and plasma cells, protecting them from oxidative damage and enhancing immune responses (167).

Selvam et al. (168) investigated the effects of vitamin E and stocking density on broiler performance and antioxidant capacity. Results showed that HSD groups given vitamin E had significant improvement in body weight, FCR, European production efficiency factor (EPEF), heterophil-to-lymphocyte (H/L) ratio, and hepatic GSH and MDA levels compared to non-supplemented HSD groups. The study concluded that adding 70 g/ton of vitamin E to HSD broiler diets effectively mitigate the adverse effects of overcrowding, enhancing both productivity and antioxidant status (168). Shehata evaluated LSD, MSD, and HSD in broilers, with vitamin E and zinc supplementation. They measured growth, hormones, gene expression, and economic outcomes. MSD and HSD impaired these parameters compared with LSD. HSD had the strongest negative effect. Vitamin E alleviated HSD-related impairments and increased profitability (169). It downregulated IL-1β, interferon-*γ*, mucin 2, and trefoil factor family 2. It also upregulated IL-4 and IL-10. Overall performance improved accordingly. These findings underscored vitamin E’s anti-inflammatory efficacy in broilers, particularly during coccidiosis vaccination-induced inflammation (170). Additional studies showed that water-soluble vitamin E (WVE) more effectively downregulated pro-inflammatory cytokine and upregulated anti-inflammatory cytokine gene expression in the jejunum compared to fat-soluble forms. Increasing WVE dosage further suppressed jejunal inflammatory markers, highlighting its superior role in modulating intestinal immune responses (171).

### 8.3 Selenomethionine (SeMet)

As an essential trace element for animals, selenium (Se) plays a vital role in maintaining organism health and antioxidant defense systems (172). This micronutrient not only participates in forming core components of antioxidant systems, but also scavenges ROS and helps to maintain redox homeostasis. The biological functions of selenium are primarily mediated through its incorporation into selenoproteins. Twenty-four selenoproteins have been identified in broilers that perform specialized roles across various cellular organelles and tissues (173). Mitochondrial selenoproteins including glutathione peroxidase 4 (GPX4), selenoprotein O (Seleno-O), and thioredoxin reductase 2 (TXNRD2) are crucial for maintaining mitochondrial function and preventing ROS-induced damage (174, 175). Endoplasmic reticulum-localized selenoproteins such as deiodinase 2 (DIO2), selenoprotein F (Seleno-F), K (Seleno-K), M (Seleno-M), N (Seleno-N), S (Seleno-S), and T (Seleno-T) play key roles in protein folding, calcium homeostasis, and cellular stress responses (176, 177). Additionally, cytoplasmic selenoproteins including glutathione peroxidase 1–3 (GPX1), GPX2, GPX3), and selenoprotein W (Seleno-W) work synergistically with their mitochondrial and endoplasmic reticulum counterparts to eliminate excess ROS, restore cellular redox balance, and protect against oxidative damage (178).

In practical poultry production, maintaining optimal Se status is critical. Deficiency impairs antioxidant defenses, increases oxidative stress susceptibility, and adversely affects growth performance and immune function. Conversely, excessive Se intake may induce selenosis characterized by growth retardation, feather loss, and neurological damage (179). Optimal Se supplementation becomes particularly important under HSD rearing or stress conditions. Organic Se forms like selenomethionine (SeMet) have enhanced bioavailability compared to inorganic sources, enabling more effective antioxidant and immunomodulatory actions (180).

Recent studies showed that SeMet effectively protected broiler liver against oxidative damage and metabolic disorders under chronic heat stress conditions. By increasing hepatic Se concentrations and upregulating key selenoproteins (GPX4, TXNRD2, etc.), SeMet enhanced antioxidant capacity while alleviating mitochondrial dysfunction, TCA cycle abnormalities, and ER stress. This treatment also helped to normalize hepatic lipid and glycogen concentrations (181). SeMet also modulates AMPK signaling pathways to inhibit lipid/glycogen synthesis while promoting their breakdown, offering potential preventive and therapeutic strategies against heat stress-induced hepatic metabolic disturbances. Comparative studies in the LMH chicken hepatoma cell line revealed superior antioxidant activity of SeMet over sodium selenite. SeMet enhanced mRNA stability and protein synthesis rates for glutathione peroxidase (GPx) and thioredoxin reductase (TrxR), and in H_2_O_2_-induced oxidative stress models, SeMet provided stronger protection through ROS/MDA/NO reduction and antioxidant enzyme activation; Nrf2 pathway activation and upregulation of antioxidant selenoenzymes further contribute to its protective effects. These findings highlight SeMet’s potential as a feed additive for preventing or mitigating oxidative damage in poultry. SeMet also exhibits significant anti-inflammatory properties. Compared to normal diets, Se supplementation significantly inhibited LPS-induced hepatic inflammatory damage by reducing oxidative stress, inflammatory cytokines, and heat shock proteins, and downregulating NLRP3 and caspase-1 expression. Mechanistically, SeMet suppresses TLR4/NF-κB/NLRP3 signaling pathways to counteract LPS-induced hepatic inflammation (182).

In conclusion, anti-inflammatory and antioxidant feed additives including CGA, VE, and SeMet show promise in mitigating HSD stress in broilers (Table 2). These compounds not only improve growth performance and feed efficiency but also enhance immune function and overall health status, effectively counteracting the negative impacts of high-density rearing on hepatic energy metabolism. Future research should focus on investigating nutrient synergies, optimizing application protocols, elucidating mechanistic pathways, and evaluating long-term efficacy and environmental impacts to develop optimal nutritional interventions.

**Table 2:** Summary of oxidative stress markers and effects of key nutritional additives in broilers under HSD.

Condition/additive	Models	Major indices	Main mechanisms	References
HSD without additive	HSD	Downregulating T-AOC, SOD, CAT, GSH-Px, GSH, ATP, complex I/III, ATPases, mitochondrial potential; Upregulating MDA, 4-HNE, ROS.	Impaired antioxidant systems; inhibited mitochondrial complexes & ATPases; lipid/protein/DNA oxidation; impaired barrier and metabolic function.	(77, 102, 106–116)
Chlorogenic acid (CGA)	HSD, oxidative or immune stress	Downregulating TNF-α, IL-1β, IL-6, MDA; Upregulating Villus height, occludin, ZO-1, SIgA, SOD, GSH-Px, CAT, T-AOC, SCFA and microbiota diversity.	Enhances barrier and mucosal immunity; activates Nrf2; modulates PPAR/MAPK; reshapes microbiota and metabolic pathways.	(161–164)
Vitamin E (VE)	HSD, Vaccination, heat stress	Downregulating MDA, normalized H/L ratio, IL-1β, IFN-γ, MUC2, TFF2; Upregulating GSH, IL-4, IL-10.	Modulating intestinal cytokines; improved immune cell function.	(165–171)
Selenomethionine (SeMet)	Chronic heat stress, LMH oxidative/inflammatory models, LPS hepatic inflammation	Downregulating ROS, MDA, NO, NLRP3, caspase-1; Upregulating hepatic Se, GPX4, TXNRD2, ER/cytosolic selenoproteins.	Supports selenoprotein network; activates Nrf2 and AMPK; suppresses TLR4/NF-κB/NLRP3 pathways; improves mitochondrial & ER function; restores hepatic antioxidant.	(172–182)

## 9 Future research gaps and perspectives

Current evidence supports that HSD exerts extensive impacts on broiler health, including oxidative stress, inflammatory responses, and metabolic disturbances. However, several limitations of the existing literature warrant attention. In particular, the mechanism of crosstalk between various organs in broilers under the HSD model is not yet clear. Future research should prioritize investigations into crosstalk mechanism between neuroendocrine, hepatic and intestinal axes (including the microbiota) in broilers under the stress model, while studies are needed to refine the dosing, timing and combinations of nutritional additives (e.g., chlorogenic acid, vitamin E and selenomethionine) to develop novel mitigation strategies to address the multifactorial challenges posed by intensive rearing conditions.

## 10 Conclusion

HSD exerts extensive impacts on broiler health, including oxidative stress, inflammatory responses, and metabolic disturbances. These effects are manifested through complex physiological mechanisms, ultimately leading to impaired growth performance and increased health complications in broiler. However, strategic implementation of specific anti-inflammatory and antioxidant feed additives including chlorogenic acid, vitamin E, and SeMet shows significant potential for mitigating the negative consequences of HSD stress. These nutritional interventions protect broiler health and enhance production performance by strengthening antioxidant defense systems, suppressing inflammatory cascades, and optimizing metabolic functions. Rational regulation of stocking density, combined with targeted use of these additives, can substantially improve both poultry welfare and economic returns in production systems. By explicitly aligning these mechanistic targets with specific nutritional interventions, this review provides a more actionable basis for mechanism-guided mitigation in intensive broiler production. Future research should prioritize investigations into optimal dosing regimens and synergistic combinations of these additives, while concurrently developing novel mitigation strategies to address the multifactorial challenges posed by intensive rearing conditions. Such advancements will be crucial for establishing sustainable poultry production practices that balance animal health with commercial productivity.
